# Prevalence of Reactive Rapid Test Results for Sexually Transmitted Infections and Associated Factors Among Transgender People Attending Specialized Health Services in Brazil

**DOI:** 10.3390/idr18050104

**Published:** 2026-09-15

**Authors:** Talia Gomes Luz, Lariane Angel Cepas, Isadora Silva de Carvalho, Alvaro Francisco Lopes de Sousa, Talita Morais Fernandes, Camila Marcheto de Sousa, Lorena Marcheto de Sousa, Ruan Nilton Rodrigues Melo, Lucas Brandão dos Santos, Mayara Souza Gomes, Lucia Alves da Silva Lara, Ariadne Ribeiro, Fatima Morales, Inês Fronteira, Ana Paula Morais Fernandes

**Affiliations:** 1 Department of General and Specialized Nursing, Ribeirão Preto School of Nursing, University of São Paulo, Ribeirão Preto 14040-902, SP, Brazil; taliagomesluz@usp.br (T.G.L.); larianeangelcepas@usp.br (L.A.C.); isadoracarvalho@usp.br (I.S.d.C.); camilamarcheto81@gmail.com (C.M.d.S.); lorenamarchetodesousa@gmail.com (L.M.d.S.); ruan.melo@usp.br (R.N.R.M.); lucas.brandao.santos@alumni.usp.br (L.B.d.S.); mayarasgomes@usp.br (M.S.G.); 2 Campus de Três Lagoas, Universidade Federal de Mato Grosso do Sul, Três Lagoas 79613-000, MS, Brazil; sousa.alvaromd@gmail.com; 3 Public Health Research Centre, Comprehensive Health Research Center (CHRC), REAL, NOVA University Lisbon, 1600-560 Lisbon, Portugal; ines.fronteira@ensp.unl.pt; 4 Higher Institute of Lisbon and Tagus Valley, 2620-379 Lisbon, Portugal; talitafernandes@usp.br; 5 Department of Gynecology and Obstetrics, Hospital das Clínicas, Ribeirão Preto Medical School, University of São Paulo, Ribeirão Preto 14048-900, SP, Brazil; luciaalvess2010@gmail.com; 6 Joint United Nations Programme on HIV/AIDS (UNAIDS), Panama City 07144, Panama; ariadnerf@gmail.com; 7 Department of Preventive Medicine and Public Health, University of Seville, 41009 Seville, Spain; fmmarin@us.es

**Keywords:** transgender people, sexually transmitted infections, HIV, sexualized substance use, transgender health, sexual health, HIV prevention, pre-exposure prophylaxis, point-of-care testing

## Abstract

Background/Objectives: Transgender (trans) individuals experience a disproportionate burden of HIV and other sexually transmitted infections (STIs), although evidence based on point-of-care testing remains limited in Brazil. This study aimed to estimate the prevalence of reactive STI rapid test results and associated factors among transgender individuals receiving care in specialized health services in São Paulo, Brazil. Methods: A cross-sectional study was conducted between July and December 2025 with 400 participants attending two specialized healthcare services. Sociodemographic, behavioral, substance use, clinical, and HIV prevention-related data were collected. The primary outcome was reactivity to at least one rapid test for HIV, syphilis, hepatitis B, or hepatitis C; reactive results could include previously known infections and therefore were not interpreted as newly diagnosed or active infections. Associations were estimated using Poisson regression with robust variance. Results: Overall, 19.2% of participants had at least one reactive rapid test result, with HIV accounting for the largest proportion of reactive results (14.8%). In the adjusted model, increasing age was associated with a higher prevalence of the outcome (PR = 1.04; 95% CI: 1.02–1.05), whereas transgender men had a lower prevalence than transgender women (PR = 0.38; 95% CI: 0.19–0.73). Reported HIV prevention through PrEP and/or PEP was inversely associated with the outcome (PR = 0.37; 95% CI: 0.17–0.82); however, this association should not be interpreted as a direct protective effect because most reactive HIV results occurred among participants with previously known HIV, who would not be eligible for PrEP. Conclusions: These findings indicate a substantial frequency of reactive STI rapid test results among transgender individuals attending specialized health services and support continued access to testing, prevention, and culturally competent sexual healthcare.

## 1. Introduction

Sexually transmitted infections (STIs) remain a major global public health concern, particularly among populations experiencing structural barriers to prevention, diagnosis, and healthcare access. Transgender (trans) individuals bear a disproportionate burden of HIV and other STIs, reflecting intersecting social vulnerabilities, institutional discrimination, economic exclusion, and inequities in access to sexual health services [1,2]. This burden is particularly pronounced among transgender women and travestis, who have substantially higher HIV prevalence than the general population [3,4]. Recent estimates suggest a global HIV prevalence of approximately 20% to 24% among transgender populations, although prevalence varies considerably across geographic and social contexts [2,5].

Beyond HIV, syphilis and viral hepatitis also represent important concerns for transgender populations [6]. Previous studies have identified multiple sexual partnerships, interconnected sexual networks, sex work, barriers to biomedical prevention, and limited access to culturally competent healthcare as relevant contexts associated with STI vulnerability [1,6]. Gender identity-related stigma may further affect access to testing and continuity of care [2]. In Brazil, these vulnerabilities coexist with social exclusion, barriers to formal employment, school dropout, and structural violence affecting transgender individuals [7]. The TransOdara study reported an HIV prevalence exceeding 34% among transgender women and travestis in Brazilian state capitals, illustrating the magnitude of HIV-related disparities in the country [8].

Changes in sexual socialization and HIV prevention have also reshaped contexts of STI exposure. Dating apps, pre-exposure prophylaxis (PrEP), post-exposure prophylaxis (PEP), and psychoactive substance use in sexual contexts have become increasingly relevant to sexual health research among key populations [9,10,11,12]. Sexualized substance use (SSU) broadly refers to the use of psychoactive substances in connection with sexual activity. This broader concept should be distinguished from classical definitions of chemsex, which generally emphasize the intentional use of specific substances, particularly methamphetamine, mephedrone, and gamma-hydroxybutyrate/gamma-butyrolactone (GHB/GBL), to facilitate, prolong, or intensify sexual experiences. Other substances, including cocaine, 3,4-methylenedioxymethamphetamine (MDMA), ketamine, poppers, cannabis, and erectile dysfunction medications, may also be used in sexual contexts [10,11,12,13].

Sexualized substance use has been associated with multiple sexual partnerships, prolonged sexual activity, inconsistent condom use, and participation in more interconnected sexual networks [9,14]. However, these associations have been studied predominantly among gay, bisexual, and other men who have sex with men, while transgender populations remain underrepresented [10,11,12,13]. Brazilian evidence also suggests that substance use, HIV, and syphilis may coexist within broader contexts of social and healthcare vulnerability [10,11,12]. Consequently, the relationship between SSU and STIs among transgender individuals should not be interpreted independently of the social and programmatic conditions in which sexual practices occur.

STI vulnerability among transgender individuals is not limited to individual behaviors. Discrimination, family rejection, economic insecurity, precarious employment, institutional barriers, and difficulties accessing appropriate prevention and treatment may interact with sexual and behavioral factors [15,16]. These dimensions are consistent with broader frameworks of structural vulnerability and syndemics, which emphasize the cumulative interaction of social, behavioral, and programmatic factors in shaping sexual health inequities [1,2].

Another limitation of the available literature is the frequent reliance on self-reported STI history or serological status [1]. Point-of-care testing can provide objective screening information during study participation and reduce dependence on participants’ previous knowledge or recall of their infection status. However, reactive rapid test results require careful clinical interpretation because they may reflect previously diagnosed HIV infection, previous or treated syphilis, or markers that do not necessarily indicate active infection. Accordingly, studies using these tests should distinguish rapid-test reactivity from newly diagnosed or active infection while considering their value for epidemiological screening and linkage to care [17,18].

Despite growing evidence on transgender sexual health, Brazilian studies simultaneously examining reactive STI rapid test results, sexual behavior, SSU, and biomedical HIV prevention among transgender individuals attending specialized healthcare services remain limited. Addressing this gap may provide a more integrated understanding of the factors associated with STI test reactivity in this service-based population. Therefore, the present study aimed to investigate the prevalence of reactive rapid test results for HIV, syphilis, hepatitis B, and hepatitis C and the factors associated with at least one reactive STI rapid test result among transgender individuals receiving care in specialized health services in Brazil.

## 2. Materials and Methods

This was a cross-sectional analytical study with a quantitative approach, conducted as a subproject of a broader multicenter investigation entitled “Projeto Pegação: Development of a Care Pathway for the Health Care of Individuals Who Practice Chemsex Within the Harm Reduction Policy Framework.”

The study included transgender (trans) individuals aged 18 years or older receiving care at two specialized public healthcare services in the state of São Paulo, Brazil. Data collection was conducted between July and December 2025. The first setting was the Reference and Training Center for STI/HIV/AIDS (CRT-SP), located in the city of São Paulo, a specialized public service for sexually transmitted infection (STI) and HIV prevention and care. The second was the Gender Incongruence Outpatient Clinic (ING) at the Hospital das Clínicas, Ribeirão Preto Medical School, University of São Paulo (HC-FMRP-USP), located in Ribeirão Preto and providing specialized multidisciplinary care for transgender individuals. Of the 400 participants included in the study, 352 (88.0%) were recruited at CRT-SP and 48 (12.0%) at HC-FMRP-USP.

Participants were recruited consecutively according to service attendance, including spontaneous attendance and internal referrals during routine clinical care. Potential participants were invited to participate in a private setting to ensure privacy and confidentiality. Data collection was conducted by previously trained members of the research team using standardized procedures. Individuals were eligible if they self-identified as transgender, were aged 18 years or older, and were receiving care at one of the participating services during the data collection period. Repeated participation was not permitted.

Rapid-test procedures and results were formally registered in the health information system as part of routine healthcare procedures, which also enabled verification that participants had not been enrolled more than once. Because recruitment was conducted among individuals attending specialized healthcare services, the study constituted a service-based, non-probability sample.

The sample size was estimated based on an approximate population of 4000 individuals registered at the participating services. The formula for estimating proportions in cross-sectional studies was applied, adopting a 95% confidence level (Z = 1.96), a 5% margin of error, and an expected proportion of 50%, a conservative value used in the absence of specific prior estimates [19]. The initial calculation resulted in a sample size of 384 participants and, after finite population correction, the minimum required sample size was estimated at 351 participants. The final sample comprised 400 participants.

Data were collected using a structured instrument administered in person and organized into sections addressing sociodemographic and economic characteristics, gender identity, sexual behavior and partnerships, psychoactive substance use in sexual contexts, HIV prevention strategies, and STI history. The instrument underwent content validation by experts in two consecutive rounds using the Content Validity Coefficient (CVC), with an overall CVC of 0.97, indicating high adequacy in terms of item clarity, relevance, and representativeness. Data were initially recorded on paper forms and subsequently entered into an electronic database, with double-entry verification procedures used for quality control.

The primary outcome was reactivity to at least one rapid screening test for HIV, syphilis, hepatitis B, or hepatitis C performed during data collection. HIV infection was assessed using the HIV GlobalX rapid cassette test (GlobalX Pharma, Hangzhou AllTest Biotech Co., Ltd., Hangzhou, China), which detects antibodies against HIV-1 and HIV-2. Syphilis was assessed using the Assure Test Syphilis (Orbitae, Assure Tech. (Hangzhou) Co., Ltd., Hangzhou, China), which detects IgG and IgM antibodies against *Treponema pallidum*. Hepatitis B was assessed using the Assure Test HBsAg (Orbitae, Assure Tech. (Hangzhou) Co., Ltd., Hangzhou, China), which detects hepatitis B surface antigen (HBsAg). Hepatitis C was assessed using the HCV GlobalX rapid cassette test (GlobalX Pharma, Hangzhou AllTest Biotech Co., Ltd., Hangzhou, China), which detects antibodies against hepatitis C virus (anti-HCV). Rapid tests were performed according to the clinical protocols of the participating services and recorded in the study database as reactive or non-reactive. Participants with previously known HIV infection were retained in the primary outcome when a reactive HIV rapid test result was recorded. Similarly, a reactive syphilis rapid test was considered part of the composite outcome regardless of previous syphilis history, recognizing that treponemal antibodies may remain detectable after adequately treated infection and that rapid-test reactivity does not necessarily indicate active syphilis. Therefore, the composite outcome represents reactivity to at least one rapid screening test and should not be interpreted as the prevalence of newly diagnosed or active infections.

For construction of the composite outcome, participants were classified as having the outcome when at least one of the available rapid test results was reactive [20]. Participants with no reactive result among the tests performed were classified as having no reactive result. For inferential analyses, the primary outcome was dichotomized as “at least one reactive STI rapid test result” and “no reactive STI rapid test result.”

The independent variables were organized into sociodemographic, sexual behavior, substance use, clinical, and prevention-related domains. Gender identity was categorized as transgender woman or transgender man according to participants’ self-identification. The number of sexual partners during the previous six months was categorized as 0, 1, or >1. An active sexual network was defined according to the availability of information on multiple sexual partnerships and/or use of dating apps to seek sexual partners. High sexual exposure was defined as the presence of multiple sexual partners, casual sex, and/or group sex. Denominators for these variables reflected the questionnaire branching logic and therefore did not necessarily represent missing data.

Consistent with previous studies, sexualized substance use was defined as the reported use of psychoactive substances before and/or during sexual activity [10,11,12]. This broader construct encompasses a wider range of substances and contexts than classical chemsex, which generally refers to the intentional use of specific psychoactive substances to enhance, prolong, or facilitate sexual experiences [11,12]. Because the substances reported in the present study extended beyond those most commonly included in classical chemsex definitions, the term sexualized substance use was adopted throughout the analyses. The separate variable ‘substance use during sex’ referred specifically to substance use during sexual activity, whereas sexualized substance use encompassed substance use either before or during sexual activity.

The specific substances reported by participants are presented in Supplementary Table S3. Polysubstance use was defined as the use of two or more psychoactive substances among participants with valid information within the sexualized substance use section of the questionnaire.

Prevention-related variables included self-reported use of pre-exposure prophylaxis (PrEP), post-exposure prophylaxis (PEP), and doxycycline post-exposure prophylaxis (doxy-PEP). These strategies were analyzed separately because of their distinct indications. The composite variable “HIV prevention” was restricted to reported PrEP and/or PEP use and did not include doxy-PEP. The variable “multiple sexual partners without PrEP/PEP” was defined as reporting more than one sexual partner during the previous six months combined with no reported PrEP or PEP use. Formal clinical eligibility or indication for PrEP was not assessed; therefore, reported PrEP use should not be interpreted as a measure of PrEP coverage among clinically eligible participants.

Statistical analyses were performed using the Statistical Package for the Social Sciences (SPSS, version 30; IBM Corp., Armonk, NY, USA). Categorical variables were summarized using absolute and relative frequencies, whereas continuous variables were described using measures of central tendency and dispersion. Bivariate analyses were conducted using participants with valid information for the variables involved in each comparison; consequently, denominators varied according to questionnaire branching, eligibility for specific questions, and availability of valid information. Pearson’s chi-square test or Fisher’s exact test was used to compare proportions, as appropriate. Crude prevalence ratios (PRs) and their respective 95% confidence intervals (95% CIs) were estimated using Poisson regression with a heteroskedasticity-consistent robust variance estimator (HC0).

Multivariable associations were estimated using Poisson regression with robust variance. Complete-case analysis was applied only to the multivariable models. The primary multivariable model included 390 of the 400 participants and 75 participants with at least one reactive STI rapid test result. Ten participants were excluded from the model: six because of invalid or inconsistent age information and four because of insufficient information to classify the variable “multiple sexual partners without PrEP/PEP.” No missing-data imputation was performed. Smaller denominators observed for several behavioral variables primarily reflected questionnaire branching or eligibility criteria rather than conventional item nonresponse.

Variables with *p* ≤ 0.20 in the bivariate analyses, together with variables considered theoretically relevant regardless of statistical significance, were considered for multivariable modeling. The initial multivariable specification considered age, race/ethnicity, gender identity, student status, entrepreneurial status, HIV prevention through PrEP and/or PEP, previous STI history, multiple sexual partners without PrEP/PEP, and sexualized substance use. Variables were evaluated according to conceptual relevance, model fit, epidemiological plausibility, and parsimony. Multicollinearity was assessed using variance inflation factors, and model fit was compared using the Akaike Information Criterion (AIC).

Previous STI history was not retained in the primary multivariable model because it is conceptually closely related to the composite rapid-test outcome and may primarily reflect cumulative disease burden and prior infection history, potentially complicating interpretation of its role as an independent explanatory factor. To assess the influence of this decision, an alternative model including previous STI history was estimated and reported as a sensitivity analysis. Because the entrepreneur category included only 13 participants, an additional sensitivity analysis excluding entrepreneurial status was performed to assess the stability of the remaining estimates.

Service site was included in descriptive and bivariate analyses. The prevalence of the primary outcome was compared between CRT-SP and HC-FMRP-USP to assess whether relevant heterogeneity was present across recruitment settings. Service site was not included in the primary multivariable model because it was not an individual-level exposure specified in the study objectives, the sample distribution between the two services was markedly unequal, and no meaningful crude difference in the primary outcome was observed between sites.

Additional sensitivity analyses were conducted to examine the influence of the composite outcome definition. These included an HIV-specific multivariable model, a descriptive analysis of HIV reactivity after excluding participants with previously known HIV-positive status, an analysis restricted to non-HIV STI rapid-test results, and an analysis restricted to participants with valid results for all four rapid tests. A separate multivariable model for syphilis was not estimated because only 20 participants had a reactive syphilis rapid test result, providing an insufficient number of events for a stable multivariable analysis. A significance level of 5% was adopted for all inferential analyses.

The study was approved by the Research Ethics Committee of the Ribeirão Preto College of Nursing, University of São Paulo (CAAE: 85545824.2.0000.5393), in accordance with Brazilian National Health Council Resolution No. 466/2012. All participants received information about the study objectives and procedures and provided written informed consent before enrollment. Privacy, confidentiality of information, and the right to withdraw at any time without affecting access to healthcare services were guaranteed throughout the study. Rapid-test procedures and results were formally recorded in the health information system in accordance with routine healthcare procedures. Participants with a reactive rapid test result were immediately referred to the appropriate healthcare service for further evaluation and clinical management according to the established protocol followed by CRT-SP and the participating services. Subsequent diagnostic and treatment information was not used to redefine the rapid-test outcome analyzed in this study.

## 3. Results

The sample consisted of 400 transgender (trans) individuals receiving care at specialized healthcare services. The mean age was 33.7 years (SD = 9.2), and 71.8% were transgender women. Most participants had 12 or more years of education (87.9%), while 66.2% reported no formal employment and 15.2% reported sex work.

Regarding sexual behavior, 76.7% of eligible participants reported more than one sexual partner in the previous six months, and 31.8% reported using dating apps to seek sexual partners. Sexualized substance use was reported by 45.8% of participants. PrEP use in the previous 12 months was reported by 17.3%, PEP use by 8.3%, and overall HIV prevention through PrEP and/or PEP by 21.3%. A previous history of STIs was reported by 44.0% of participants, and 19.2% had at least one reactive rapid test result for HIV, syphilis, hepatitis B, or hepatitis C (Table 1).

As shown in Table 2, 19.2% of participants had at least one reactive rapid test result in the primary analysis. Because only 270 participants had valid results for all four rapid tests, a complete-testing sensitivity analysis was conducted; among these participants, 43 of 270 (15.9%) had at least one reactive result. HIV showed the highest proportion of reactive results (14.8%), followed by syphilis (7.4%), hepatitis B (0.8%), and hepatitis C (0.5%).

Sensitivity analyses showed that, after excluding 52 participants with previously known HIV-positive status, 10 of 348 participants (2.9%) had a reactive HIV rapid test result. When HIV was excluded from the composite outcome, 23 of 400 participants (5.8%) had at least one reactive non-HIV STI rapid test result. Syphilis reactivity was observed in 20 of 271 tested participants (7.4%). Multivariable modeling for syphilis was not performed because of the limited number of reactive results. In an additional sensitivity analysis restricted to the 270 participants with valid results for all four rapid tests, 43 (15.9%) had at least one reactive result.

As shown in Table 3, transgender men had a lower prevalence of reactive STI test results than transgender women (8.8% vs. 23.3%; PR = 0.38; 95% CI: 0.20–0.71). Higher prevalence was also observed among Black participants compared with White participants (PR = 1.78; 95% CI: 1.05–3.01) and among entrepreneurs compared with non-entrepreneurs (PR = 2.52; 95% CI: 1.35–4.69).

Previous STI history was strongly associated with the outcome (PR = 10.98; 95% CI: 5.42–22.21), as was having multiple sexual partners without PrEP/PEP (PR = 1.96; 95% CI: 1.31–2.91). Conversely, PrEP use (PR = 0.41; 95% CI: 0.18–0.89) and HIV prevention through PrEP and/or PEP (PR = 0.37; 95% CI: 0.18–0.78) were associated with a lower prevalence of reactive STI test results. Sexualized substance use was not significantly associated with the outcome.

After the initial modeling, previous STI history was excluded from the primary multivariable model because of its close conceptual relationship with the composite outcome and its potential to represent cumulative disease burden rather than an independent exposure. This decision was not based on improved model fit; the model including previous STI history showed a lower AIC and is presented as a sensitivity analysis.

The final model included 390 participants and 75 reactive outcomes. Increasing age was associated with a higher prevalence of at least one reactive STI rapid test result (adjusted PR = 1.04; 95% CI: 1.02–1.05), whereas transgender men had a lower prevalence than transgender women (adjusted PR = 0.38; 95% CI: 0.19–0.73). An exploratory association was observed for entrepreneurial status (adjusted PR = 2.87; 95% CI: 1.82–4.50); however, this estimate was based on only 13 participants, including six with a reactive outcome, and is therefore potentially unstable and should be interpreted cautiously. HIV prevention through PrEP and/or PEP was inversely associated with the outcome (adjusted PR = 0.37; 95% CI: 0.17–0.82) (Table 4).

## 4. Discussion

The present study identified a substantial proportion of reactive sexually transmitted infection (STI) rapid test results among transgender individuals receiving care in specialized health services in Brazil. Overall, 19.2% of participants had at least one reactive rapid test result, with HIV accounting for most reactive results. Transgender men had a lower prevalence of the composite outcome than transgender women, while increasing age and entrepreneurial status were positively associated with reactivity in the primary adjusted model. Reported use of HIV prevention through pre-exposure prophylaxis (PrEP) and/or post-exposure prophylaxis (PEP) was inversely associated with the outcome.

The proportion of participants with at least one reactive rapid test result is consistent with previous evidence showing a substantial burden of HIV and other STIs among transgender populations, particularly transgender women and travestis [4,6]. However, the composite outcome requires cautious interpretation because it included previously diagnosed infections. HIV rapid-test reactivity was observed in 14.8% of participants, but 49 of the 59 participants with a reactive HIV result had previously reported living with HIV. Therefore, the HIV component primarily reflects prevalent HIV infection rather than newly acquired infection. Similarly, syphilis rapid-test reactivity cannot be interpreted as active syphilis in all cases because treponemal antibodies may remain detectable after previously treated infection. These characteristics limit direct comparisons with studies reporting incident, newly diagnosed, or laboratory-confirmed active infections.

Within the Brazilian context, the findings are broadly consistent with the TransOdara study, which reported a high prevalence of HIV among transgender women and travestis in Brazilian state capitals [8]. The lower HIV rapid-test reactivity observed in the present study may partly reflect differences in study populations, particularly the inclusion of both transgender women and transgender men and the recruitment of participants already engaged with specialized healthcare services. Because the current sample was service-based, the findings should not be interpreted as representative of transgender people in Brazil, particularly those experiencing greater barriers to healthcare access.

The use of point-of-care testing represents a strength because it provided objective screening results at the time of study participation rather than relying exclusively on self-reported STI status. Previous research has shown that some transgender individuals may be unaware of their HIV status, particularly in contexts of limited or inconsistent healthcare access [21]. Nevertheless, rapid-test reactivity should be distinguished from confirmation of newly diagnosed or active infection, as emphasized by the sensitivity analyses conducted in the present study.

Differences according to gender identity were also observed. Transgender men had a lower prevalence of reactive STI rapid test results than transgender women, consistent with previous literature documenting important disparities in HIV and STI burden between transgender populations [3,4]. However, the present study did not assess sufficiently detailed sexual practices, partner gender, anatomy-specific exposure, condom use according to sexual practice, or other factors that could explain biological differences in exposure. Accordingly, the observed association should not be attributed to specific sexual practices or partnership patterns that were not directly measured. Broader structural and social inequalities affecting transgender women remain relevant interpretative contexts in the literature [1], including differences in social exclusion, violence, healthcare access, and participation in sexual networks with a high prevalence of HIV and other STIs [22]. These mechanisms, however, were not directly tested in the present study.

Although transgender men had a lower prevalence of the outcome, this finding should not be interpreted as indicating absence of vulnerability. Transgender men remain underrepresented in epidemiological studies of HIV and other STIs, which may contribute to important gaps in knowledge, testing strategies, and prevention approaches [4]. Further research specifically designed to characterize sexual practices, partner characteristics, prevention needs, and anatomy-relevant exposures among transgender men is warranted.

Increasing age remained associated with reactive rapid test results in the adjusted analysis. This association should be interpreted cautiously because the cross-sectional outcome included previously diagnosed chronic HIV infection and potentially previously treated syphilis. Thus, the association with age may partly reflect cumulative lifetime exposure and longer duration of prevalent infection rather than increased recent STI acquisition. Older transgender individuals may also have experienced periods characterized by more limited HIV testing, prevention technologies, and access to affirming healthcare [23]. However, the present design cannot distinguish among these potential explanations.

Entrepreneurial status was also associated with a higher prevalence of the outcome. This result warrants particular caution because only 13 participants were classified as entrepreneurs, of whom six had a reactive STI rapid test result. The estimate is therefore based on a sparse category, is potentially unstable, and should not be interpreted as evidence that entrepreneurship itself increases STI vulnerability. Although precarious self-employment and labor-market exclusion have been discussed in studies of marginalized populations [24], and employment inequalities have been documented among transgender people in Brazil [8], the present study did not collect sufficiently detailed information on the nature, stability, or socioeconomic characteristics of entrepreneurial activities to support a specific explanatory mechanism. Importantly, sensitivity analysis excluding this variable did not materially alter the estimates of the remaining predictors.

Previous STI history showed the strongest crude association with the outcome. Because previous STI occurrence may represent cumulative exposure and also overlaps conceptually with an outcome that includes prevalent and previously diagnosed infections, it was not retained in the primary multivariable model. Nevertheless, a sensitivity model including previous STI history was performed and showed a strong association with the outcome, while also improving statistical fit. The difference between models emphasizes that previous STI history should be viewed as an important marker of cumulative sexual health history rather than as a straightforward independent exposure [24,25]. These findings should therefore be interpreted within the limitations of the cross-sectional design rather than causally.

Sexualized substance use was common but was not independently associated with the composite outcome. Previous studies have documented sexualized substance use among sexual and gender minority populations, including Brazilian transgender populations [10,26,27,28], but definitions and substances included vary substantially across studies. In the present study, the operational definition was intentionally broader than classical definitions of chemsex and included a range of psychoactive substances used before or during sexual activity [26,27,28]. Accordingly, the absence of an independent association should be interpreted specifically in relation to this broader measure rather than as evidence regarding classical chemsex. The underrepresentation of transgender populations in this literature also remains an important research gap [24,25,26,27,28].

Reported use of biomedical HIV prevention was relatively uncommon in the overall sample, with 17.3% reporting PrEP and 8.3% reporting PEP. These proportions should not be interpreted as measures of inadequate PrEP coverage because clinical eligibility or indication for PrEP was not assessed. Previous studies have nevertheless identified barriers to PrEP access and uptake among transgender populations in Brazil [29,30]. The inverse association observed between PrEP/PEP use and the composite outcome also requires cautious interpretation. Because most reactive HIV results occurred among participants already known to be living with HIV, who would not be candidates for PrEP, part of this association may reflect differences in prevention indication and HIV status rather than a direct protective effect of PrEP or PEP on the composite outcome.

More broadly, transgender individuals may experience barriers to healthcare related to discrimination, inadequate professional training, fragmentation of gender-affirming and sexual healthcare, and difficulties sustaining engagement in prevention services [31,32,33]. These structural factors are well documented in the literature [34,35,36,37], but they were not directly measured as causal determinants of reactive STI results in the present study. Consequently, they should be understood as relevant interpretative context rather than as mechanisms demonstrated by our findings.

Taken together, the findings indicate that reactive STI rapid test results were common in this service-based sample and that their distribution differed according to gender identity, age, and selected sociodemographic and prevention-related characteristics. The results also highlight the importance of distinguishing prevalent or previously diagnosed infections from newly acquired infections when interpreting composite STI outcomes. Rather than attributing the observed associations to unmeasured behavioral or structural mechanisms, the findings support the need for further longitudinal and community-based research incorporating detailed sexual practices, infection-specific outcomes, prevention eligibility, and social and healthcare determinants among diverse transgender populations.

## 5. Limitations

The cross-sectional design of this study precludes causal inference and does not allow differentiation between recent STI acquisition and previously diagnosed or treated infections. In particular, the composite outcome included reactive rapid test results that could reflect known HIV infection or previous syphilis, and therefore should not be interpreted as the prevalence of newly diagnosed or active infections. Participants were recruited consecutively from two specialized public healthcare services in the state of São Paulo, resulting in a service-based, non-probability sample. Accordingly, the findings primarily reflect transgender individuals already engaged with specialized healthcare and should not be generalized to the broader transgender population in Brazil, particularly those facing greater barriers to healthcare access.

Only 270 of the 400 participants had valid results for all four rapid tests. Participants without a reactive result among the tests performed were classified as non-reactive for the composite outcome even when one or more test results were unavailable. Accordingly, incomplete testing may have influenced the primary estimate of 19.2%; however, the sensitivity analysis restricted to participants with complete testing yielded a prevalence of 15.9%.

Sexual behavior and substance use variables were self-reported and may be subject to recall and social desirability bias. In addition, the study did not collect sufficiently detailed information on specific sexual practices, partner gender, anatomy-related exposure, or other factors that could explain differences in biological exposure between transgender women and transgender men. Differences in denominators across some variables largely reflected questionnaire branching and eligibility criteria, although some missing information was also present.

## 6. Conclusions

This study identified a substantial frequency of reactive STI rapid test results among transgender individuals receiving care in specialized healthcare services in São Paulo, Brazil, with HIV accounting for most reactive results and a lower prevalence observed among transgender men than transgender women. Because the composite outcome included previously known HIV infection and potentially previously treated syphilis, these findings should be interpreted as rapid-test reactivity rather than as the prevalence of newly diagnosed or active infections.

Increasing age was associated with a higher prevalence of the composite outcome, although this association may partly reflect cumulative lifetime exposure and the contribution of prevalent HIV infection. Reported HIV prevention through PrEP and/or PEP was inversely associated with the outcome, but this finding should be interpreted cautiously because PrEP eligibility was not assessed and most reactive HIV results occurred among participants already known to be living with HIV.

These findings may help inform the organization of rapid testing, HIV prevention strategies, and culturally competent sexual healthcare within specialized services. Longitudinal and community-based studies incorporating infection-specific outcomes, detailed sexual exposure measures, prevention eligibility, and broader social and healthcare determinants are needed to further characterize STI vulnerability across diverse transgender populations.

## Figures and Tables

**Table 1:** Sociodemographic, behavioral, prevention-related, and substance use characteristics of transgender individuals participating in the study (N = 400).

Variable	n	%
Sociodemographic Characteristics		
Age (years) (n = 394)		
Mean (SD)	33.7 (9.2)	—
Median	32	—
Min–Max	18–63	—
Race/Ethnicity (N = 400)		
White	179	44.8
Mixed-race	144	36.0
Black	67	16.8
Other	10	2.5
Gender Identity (N = 400)		
Transgender woman	287	71.8
Transgender man	113	28.2
Service site		
CRT-SP	352	88
HCFMRP	48	12
Education (N = 398)		
<12 years	48	12.1
≥12 years	350	87.9
Monthly Income (N = 379)		
Up to one minimum wage	142	37.5
Two or more minimum wages	237	62.5
Occupational Status		
Student (N = 400)		
Yes	52	13.0
No	348	87.0
Formal worker (N = 400)		
Yes	135	33.8
No	265	66.2
Informal worker (N = 400)		
Yes	156	39.0
No	244	61.0
Self-employed worker (N = 400)		
Yes	110	27.5
No	290	72.5
Unemployed with financial assistance (N = 400)		
Yes	13	3.2
No	387	96.8
Unemployed without financial assistance (N = 400)		
Yes	30	7.5
No	370	92.5
Entrepreneur (N = 400)		
Yes	13	3.2
No	387	96.8
Sex work (N = 400)		
Yes	61	15.2
No	339	84.8
Sexual Behavior and Networks		
Number of sexual partners (past 6 months) (N = 262)		
0	27	10.3
1	34	13.0
>1	201	76.7
Type of sexual partnership (N = 388)		
Steady	133	34.3
Casual	134	34.5
Both (steady and casual)	121	31.2
Use of dating apps (N = 400)		
Yes	127	31.8
No	273	68.2
Active sexual network (N = 273)		
Yes	226	82.8
No	47	17.2
Substance Use–Related Variables		
Substance use during sex in the past 12 months (N = 400)		
Yes	182	45.5
No	218	54.5
Sexualized substance use (N = 400)		
Yes	183	45.8
No	217	54.2
Polysubstance use (N = 181)		
Yes	103	56.9
No	78	43.1
Prevention Variables		
PrEP use (past 12 months) (N = 400)		
Yes	69	17.3
No	331	82.8
PEP use (past 12 months) (N = 400)		
Yes	33	8.3
No	367	91.8
Previous use of doxy-PEP (N = 400)		
Yes	19	4.8
No	381	95.2
HIV prevention (PrEP/PEP) (N = 400)		
Yes	85	21.3
No	315	78.8
Clinical Variables Related to STIs		
Previous history of STIs (N = 400)		
Yes	176	44.0
No	224	56.0
Reactive rapid test result * (N = 400)		
Yes	77	19.2
No	323	80.8

Note: Values are expressed as absolute frequencies (n) and percentages (%); SD, standard deviation; PrEP, pre-exposure prophylaxis; PEP, post-exposure prophylaxis; STI, sexually transmitted infection. Variable-specific denominators reflect questionnaire branching, eligibility criteria, or availability of valid information. The number of sexual partners was assessed among 262 eligible participants according to the questionnaire branching logic; active sexual network status could be classified for 273 participants; and polysubstance use was assessed among 181 participants with valid information within the sexualized substance use block. * The variable “Reactive rapid test result” corresponds to at least one reactive rapid test result for HIV, syphilis, hepatitis B, or hepatitis C obtained during data collection.

**Table 2:** Reactive rapid test result among transgender individuals participating in the study.

Indicator	Reactive (n)	Non-Reactive (n)	Not Performed/Not Reported (n)	Total Tested (N)	Prevalence (%)
Reactive result for ≥1 STI	77	323	—	400	19.2
HIV	59	340	1	399	14.8
Syphilis	20	251	129	271	7.4
Hepatitis B	3	397	0	400	0.8
Hepatitis C	2	398	0	400	0.5

Note: Prevalence for each infection was calculated using the number of participants with a valid rapid test result as the denominator. For HIV, 399 participants had a valid result and one test was recorded as not performed/not reported. For syphilis, 271 participants had a valid rapid test result, whereas 129 had no valid result recorded; non-testing occurred predominantly among participants with a previous history of syphilis. Hepatitis B and hepatitis C rapid test results were available for all 400 participants. The composite outcome “reactive result for ≥1 STI” was defined as at least one reactive rapid test result for HIV, syphilis, hepatitis B, or hepatitis C among the tests performed during data collection. A non-reactive classification for the composite outcome indicates that no reactive result was identified among the rapid tests performed and does not imply that all four tests were necessarily performed for every participant. Among the 270 participants with valid results for all four rapid tests, 43 (15.9%) had at least one reactive result.

**Table 3:** Bivariate analysis of sociodemographic, behavioral, clinical, and prevention-related factors associated with reactive rapid test result for sexually transmitted infections (STIs).

Variables	n	%	*p*-Value	PR	95% CI
Sociodemographic Characteristics					
Race/Ethnicity (N = 400; *p*-global = 0.146)					
White	27	15.1	—	1.00	—
Other	3	30.0	0.181	1.99	0.73–5.45
Mixed-race	29	20.1	0.234	1.34	0.83–2.15
Black	18	26.9	0.032	1.78	1.05–3.01
Gender Identity (N = 400; *p*-global = 0.002)					
Transgender Woman	67	23.3	—	1.00	—
Transgender man	10	8.8	0.002	0.38	0.20–0.71
Service Site (N = 400; *p*-global = 0.765)					
CRT-SP	67	19.0	—	1.00	—
HCFMRP	10	20.8	0.765	1.09	0.61–1.98
Education (N = 398; *p*-global = 0.742)					
<12 years	10	20.8	—	1.00	—
≥12 years	66	18.9	0.742	0.91	0.50–1.64
Monthly Income (N = 379; *p*-global = 0.870)					
Two or more minimum wages	45	19.0	—	1.00	—
Up to one minimum wage	26	18.3	0.870	0.96	0.62–1.49
Occupational Status					
Student (N = 400; *p*-global = 0.080)					
No	72	20.7	—	1.00	—
Yes	5	9.6	0.080	0.46	0.20–1.10
Formal worker (N = 400; *p*-global = 0.427)					
No	54	20.4	—	1.00	—
Yes	23	17.0	0.427	0.84	0.54–1.30
Self-employed worker (N = 400; *p*-global = 0.814)					
No	55	19.0	—	1.00	—
Yes	22	20.0	0.814	1.05	0.68–1.64
Sex work (N = 400; *p*-global = 0.418)					
No	63	18.6	—	1.00	—
Yes	14	23.0	0.418	1.23	0.74–2.06
Unemployed with financial assistance (N = 400; *p*-global = 0.716)					
No	74	19.1	—	1.00	—
Yes	3	23.1	0.716	1.21	0.44–3.32
Unemployed without financial assistance (N = 400; *p*-global = 0.913)					
No	71	19.2	—	1.00	—
Yes	6	20.0	0.913	1.04	0.49–2.20
Entrepreneur (N = 400; *p*-global = 0.004)					
No	71	18.3	—	1.00	—
Yes	6	46.2	0.004	2.52	1.35–4.69
Informal worker (N = 400; *p*-global = 0.439)					
No	44	18.0	—	1.00	—
Yes	33	21.2	0.439	1.17	0.78–1.76
Sexual Behavior and Network Characteristics					
Number of sexual partners (past 6 months) (N = 262; *p*-global = 0.130)					
>1	41	20.4	—	1.00	—
0	1	3.7	0.085	0.18	0.03–1.27
1	4	11.8	0.261	0.58	0.22–1.51
Type of sexual partnership (N = 388; *p*-global = 0.289)					
Both (steady and casual)	18	14.9	—	1.00	—
Casual	28	20.9	0.216	1.40	0.82–2.41
Steady	30	22.6	0.124	1.52	0.89–2.58
Active sexual network (N = 273; *p*-global = 0.096)					
No	4	8.5	—	1.00	—
Yes	44	19.5	0.096	2.29	0.86–6.06
Use of dating apps (N = 400; *p*-global = 0.233)					
No	57	20.9	—	1.00	—
Yes	20	15.7	0.233	0.75	0.47–1.20
High sexual exposure (N = 394; *p*-global = 0.281)					
No	31	22.5	—	1.00	—
Yes	46	18.0	0.281	0.80	0.53–1.20
Substance Use-Related Variables					
Sexualized substance use (N = 400; *p*-global = 0.225)					
No	37	17.1	—	1.00	—
Yes	40	21.9	0.225	1.28	0.86–1.92
Substance use during sex in the past 12 months (N = 400; *p*-global = 0.207)					
No	37	17.0	—	1.00	—
Yes	40	22.0	0.207	1.29	0.87–1.93
Polysubstance use (N = 181; *p*-global = 0.422)					
No	15	19.2	—	1.00	—
Yes	25	24.3	0.422	1.26	0.71–2.23
Prevention and Clinical Variables					
PrEP use (N = 400; *p*-global = 0.025)					
No	71	21.5	—	1.00	—
Yes	6	8.7	0.025	0.41	0.18–0.89
PEP use (N = 400; *p*-global = 0.155)					
No	74	20.2	—	1.00	—
Yes	3	9.1	0.155	0.45	0.15–1.35
Doxy-PEP use (N = 400; *p*-global = 0.837)					
No	73	19.2	—	1.00	—
Yes	4	21.1	0.837	1.10	0.45–2.69
HIV prevention (PrEP/PEP) (N = 400; *p*-global = 0.008)					
No	70	22.2	—	1.00	—
Yes	7	8.2	0.008	0.37	0.18–0.78
Previous history of STIs (N = 400; *p*-global < 0.001)					
No	8	3.6	—	1.00	—
Yes	69	39.2	<0.001	10.98	5.42–22.21
Social isolation (N = 400; *p*-global = 0.396)					
No	20	16.7	—	1.00	—
Yes	57	20.4	0.396	1.22	0.77–1.94
Multiple sexual partners without PrEP/PEP (N = 396; *p*-global < 0.001)					
No	38	14.5	—	1.00	—
Yes	38	28.4	<0.001	1.96	1.31–2.91

Note: n represents the number of participants with at least one reactive sexually transmitted infection rapid test result within each category, and % represents the corresponding prevalence. Crude prevalence ratios (PRs), 95% confidence intervals (95% CIs), and *p*-values for individual contrasts were estimated using Poisson regression with robust variance (HC0). For variables with more than two categories, the overall significance of the factor was assessed using the robust Wald test and is reported as the *p*-global value. The reference category is indicated by PR = 1.00. Denominators vary according to questionnaire eligibility and the availability of valid information. For “multiple sexual partners without PrEP/PEP,” the valid denominator was 396 because four participants could not be classified. For polysubstance use, the valid denominator was 181 because two participants within the sexualized substance use group selected “Prefer not to answer” and were treated as missing for this variable.

**Table 4:** Factors associated with reactive rapid test result after exclusion of previous STI history from the model.

Variables	Reactive n/N (%)	Crude PR	95% CI	*p*-Value	Adjusted PR	95% CI	*p*-Value
Sociodemographic Characteristics							
Age (years)	-	1.04	1.02–1.06	<0.001	1.04	1.02–1.05	<0.001
Gender Identity							
Transgender woman	65/279 (23.3%)	1.00	Reference		1.00	Reference	
Transgender man	10/111 (9.0%)	0.38	0.20–0.71	0.002	0.38	0.19–0.73	0.004
Occupational Status							
Entrepreneur							
No	69/377 (18.3%)	1.00	Reference		1.00	Reference	
Yes	6/13 (46.2%)	2.52	1.35–4.69	0.004	2.87	1.82–4.50	<0.001
Prevention and Sexual Behavior							
HIV prevention (PrEP/PEP)							
No	68/307 (22.1%)	1.00	Reference		1.00	Reference	
Yes	7/83 (8.4%)	0.37	0.18–0.78	0.008	0.37	0.17–0.82	0.014
Multiple sexual partners without PrEP/PEP							
No	38/259 (14.7%)	1.00	Reference		1.00	Reference	
Yes	37/131 (28.2%)	1.96	1.31–2.91	<0.001	1.33	0.86–2.05	0.198

Note: The final model was estimated using Poisson regression with robust variance and included 390 participants, of whom 75 had at least one reactive sexually transmitted infection rapid test result. PR = prevalence ratio; 95% CI = 95% confidence interval; PrEP = pre-exposure prophylaxis; PEP = post-exposure prophylaxis. Transgender woman and “No” were used as the reference categories. Previous STI history was excluded from the final model on conceptual and parsimony grounds rather than because its exclusion improved model fit. The final model had an Akaike Information Criterion (AIC) of 372.87. Variance inflation factors ranged from 1.05 to 1.26, indicating no evidence of problematic multicollinearity.

## Data Availability

The datasets generated and analyzed during the current study are available from the corresponding author upon reasonable request, subject to ethical and institutional restrictions.

## Supplementary Materials

The following supporting information can be downloaded at: https://www.mdpi.com/article/10.3390/idr18050104/s1, Table S1. Multivariable analysis of factors associated with at least one reactive sexually transmitted infection rapid test result, including previous STI history; Table S2. Sensitivity analyses of factors associated with reactive sexually transmitted infection rapid test results; Table S3. Psychoactive substances reported among participants classified as having sexualized substance use (N = 183).

## Abbreviations

The following abbreviations are used in this manuscript:

AIC	Akaike Information Criterion
AIDS	Acquired Immunodeficiency Syndrome
CAAE	Certificate of Presentation for Ethical Consideration (Certificado de Apresentação para Apreciação Ética)
CAPES	Coordination for the Improvement of Higher Education Personnel (Coordenação de Aperfeiçoamento de Pessoal de Nível Superior)
CRT	Reference and Training Center for STI/HIV/AIDS (Centro de Referência e Treinamento em IST/HIV/AIDS)
CVC	Content Validity Coefficient
GHB	Gamma-Hydroxybutyrate
GBL	Gamma-Butyrolactone
HC-FMRP-USP	Hospital das Clínicas, Ribeirão Preto Medical School, University of São Paulo
HIV	Human Immunodeficiency Virus
ING	Gender Incongruence Outpatient Clinic
MDMA	3,4-Methylenedioxymethamphetamine
PEP	Post-Exposure Prophylaxis
PR	Prevalence Ratio
PrEP	Pre-Exposure Prophylaxis
SD	Standard Deviation
SPSS	Statistical Package for the Social Sciences
STI	Sexually Transmitted Infection
SUS	Unified Health System (Sistema Único de Saúde)
95% CI	95% Confidence Interval
