# Supplementary material for: Prevalence of Reactive Rapid Test Results for Sexually Transmitted Infections and Associated Factors Among Transgender People Attending Specialized Health Services in Brazil
Source: Infect Dis Rep. 2026 Sep 15;18(5):104. doi: 10.3390/idr18050104 (PMC13600157; doi:10.3390/idr18050104)
Supplement: Supplementary file 1 [file idr-18-00104-s001.zip › idr-4419544-supplementary.pdf]

Supplementary Materials

Supplementary Table S1. Multivariable analysis of factors associated with at least one reactive sexually transmitted infection rapid test result, including previous STI history.

Variables	Crude PR	95% CI	p-value	Adjusted PR	95% CI	p-value
Age (years)	1.04	1.02–1.06	<0.001	1.02	1.01–1.04	0.007
Race/Ethnicity						
White	1.00	Reference	—	1.00	Reference	—
Other	1.99	0.73–5.45	0.181	1.22	0.60–2.50	0.578
Mixed-race	1.34	0.83–2.15	0.234	0.99	0.66–1.49	0.955
Black	1.78	1.05–3.01	0.032	1.16	0.76–1.78	0.483
Gender Identity						
Transgender woman	1.00	Reference	—	1.00	Reference	—
Transgender man	0.38	0.20–0.71	0.002	1.03	0.57–1.86	0.917
Student						
No	1.00	Reference	—	1.00	Reference	—
Yes	0.46	0.20–1.10	0.080	0.79	0.37–1.69	0.548
Entrepreneur						
No	1.00	Reference	—	1.00	Reference	—
Yes	2.52	1.35–4.69	0.004	2.10	1.41–3.13	<0.001
HIV prevention (PrEP/PEP)						
No	1.00	Reference	—	1.00	Reference	—
Yes	0.37	0.18–0.78	0.008	0.26	0.12–0.57	<0.001
Previous history of STIs						
No	1.00	Reference	—	1.00	Reference	—
Yes	10.98	5.42–22.21	<0.001	11.56	5.22–25.57	<0.001
Multiple sexual partners without PrEP/PEP						
No	1.00	Reference	—	1.00	Reference	—
Yes	1.96	1.31–2.91	<0.001	0.79	0.53–1.17	0.242
Sexualized substance use						
No	1.00	Reference	—	1.00	Reference	—
Yes	1.28	0.86–1.92	0.225	1.02	0.72–1.45	0.914

Note: The model was estimated using Poisson regression with robust variance and included 390 participants, of whom 75 had at least one reactive sexually transmitted infection rapid test result. PR = prevalence ratio; 95% CI = 95% confidence interval; PrEP = pre-exposure prophylaxis; PEP = post-exposure prophylaxis. AIC = 329.88. Previous STI history was retained in this sensitivity model to assess the influence of cumulative prior STI occurrence on the estimates obtained in the primary model. The lower AIC of this model indicates better statistical fit; however, previous STI history was excluded from the primary model for conceptual and parsimony reasons.

Supplementary Table S2. Sensitivity analyses of factors associated with reactive sexually transmitted infection rapid test results.

Panel A. Multivariable model with reactive HIV rapid test result as the outcome

Variables	Adjusted PR	95% CI	p-value
Age (years)	1.04	1.02–1.06	<0.001
Gender Identity			
Transgender woman	1.00	Reference	—
Transgender man	0.23	0.09–0.58	0.002
Entrepreneur			
No	1.00	Reference	—
Yes	3.33	1.98–5.59	<0.001
HIV prevention (PrEP/PEP)			
No	1.00	Reference	—
Yes	0.20	0.06–0.65	0.008
Multiple sexual partners without PrEP/PEP			
No	1.00	Reference	—
Yes	1.43	0.86–2.36	0.167

Note: Poisson regression with robust variance; N = 389; 57 reactive HIV test results; AIC = 298.73.

Panel B. Sensitivity analysis excluding entrepreneur from the primary multivariable model

Variables	Primary model Adjusted PR (95% CI)	p-value	Model without entrepreneur Adjusted PR (95% CI)	p-value
Age (years)	1.04 (1.02–1.05)	<0.001	1.04 (1.02–1.05)	<0.001
Transgender man	0.38 (0.19–0.73)	0.004	0.41 (0.21–0.79)	0.007
HIV prevention (PrEP/PEP)	0.37 (0.17–0.82)	0.014	0.39 (0.18–0.85)	0.017
Multiple sexual partners without PrEP/PEP	1.33 (0.86–2.05)	0.198	1.43 (0.93–2.19)	0.101

Note: Both models included the same 390 participants and 75 outcome events. The primary model had an AIC of 372.87, whereas the model excluding entrepreneur had an AIC of 375.36. The similarity of the estimates indicates that exclusion of the entrepreneur variable did not materially change the direction or interpretation of the remaining associations.

Panel C. Descriptive sensitivity analyses according to HIV status and infection type

Sensitivity Analysis	N analyzed	Reactive, n	Prevalence (%)
At least one reactive STI rapid test, primary analysis	400	77	19.2
HIV rapid test	399	59	14.8
HIV rapid test after excluding participants with previously known HIV-positive status	348	10	2.9
At least one reactive non-HIV STI rapid test	400	23	5.8
Syphilis	271	20	7.4
Hepatitis B	400	3	0.8
Hepatitis C	400	2	0.5
Complete testing for all four infections	270	43	15.9

Note: Fifty-two participants self-reported a previous HIV diagnosis. Of these, 49 had a reactive HIV rapid test during data collection, whereas three did not have a reactive rapid-test result recorded. Previous HIV diagnosis and rapid-test reactivity were treated as distinct variables. Among the 59 participants with a reactive HIV rapid test during data collection, 49 (83.1%) belonged to this group. Among the remaining 10 participants with reactive HIV tests, seven previously reported an HIV-negative status, two did not know their status, and one preferred not to report it. Therefore, these 10 participants should not necessarily be interpreted as newly diagnosed HIV cases. Multivariable models were not estimated after excluding participants with previously known HIV or for non-HIV STIs because of the limited number of outcome events.

Supplementary Table S3. Psychoactive substances reported among participants classified as having sexualized substance use (N = 183).

**Substance**	**n**	**%**
Alcohol	155	84.7
Cannabis	108	59.0
Cocaine	56	30.6
MDMA/Ecstasy	28	15.3
Sildenafil/tadalafil or similar medications	25	13.7
Inhaled ether-based recreational substances (“lança-perfume/loló”)	23	12.6
Ketamine	14	7.7
LSD	12	6.6
Crack cocaine	11	6.0
GHB/GBL	10	5.5
Poppers	10	5.5
Methamphetamine/crystal meth	9	4.9
Psilocybin/mushrooms	7	3.8
Benzodiazepines	3	1.6
Other[1]	2	1.1
Mephedrone	1	0.5
Heroin	1	0.5

Note: Percentages were calculated among the 183 participants classified as having sexualized substance use. Multiple responses were permitted; therefore, percentages do not sum to 100%. [1]Other substances included tobacco (n = 1) and ayahuasca (n = 1). Sexualized substance use was operationalized as the reported use of psychoactive substances before or during sexual activity during the previous 12 months.
